# MicroRNA-325-3p protects the heart after myocardial infarction by inhibiting RIPK3 and programmed necrosis in mice

**DOI:** 10.1186/s12867-019-0133-z

**Published:** 2019-06-27

**Authors:** Dong-Ying Zhang, Bing-Jian Wang, Min Ma, Kun Yu, Qing Zhang, Xi-Wen Zhang

**Affiliations:** 1 Department of Cardiology, The Affiliated Huaian No.1 People’s Hospital of Nanjing Medical University, No.1 West Huanghe Road, Huaiyin District, Huaian, 223300 Jiangsu China; 2 Department of Cardiology, The Sixth People’s Hospital of Chengdu, Chengdu, 610051 China

**Keywords:** Myocardial infarction, Necroptosis, RIPK3, MiR-325-3p

## Abstract

**Background:**

Receptor-interacting serine-threonine kinase 3 (RIPK3)-mediated necroptosis has been implicated in the progression of myocardial infarction (MI), but the underlying mechanisms, particularly whether microRNAs (miRNAs) are involved, remain largely unknown.

**Results:**

A microarray analysis was used to screen for miR-325-3p expression in myocardial tissues from MI mice, and the expression was confirmed with qRT-PCR. The levels of myocardial enzymes were measured using commercial kits, and an echocardiography system was utilized for the detection of cardiac function parameters. The pathological features and infarction sizes of cardiac tissues were examined using H&E, TCC and Masson’s trichrome staining, and the amount of cell apoptosis was determined using an in situ TUNEL assay. Cardiomyocytes were isolated and then subjected to hypoxia induction in vitro. The expression of the RIPK1, RIPK3 and phosphorylated MLKL (p-MLKL) proteins was measured using a Western blot. The mouse cardiomyocyte cell viability was analyzed by an MTT assay. The mRNA target of miR-325-3p was predicted using TargetScan v7.2 and then validated using a dual-luciferase reporter assay. The overexpression of miR-325-3p evidently decreased the expression levels of lactate dehydrogenase (LDH), phosphocreatine kinase (CK), superoxide dismutase (SOD) and malondialdehyde (MDA), inhibited left ventricular end-diastolic diameter (LVEDD) and left ventricular end-systolic diameter (LVESD), and promoted left ventricular ejection fraction (LVEF) and left ventricular fractional shortening (LVES). In addition, miR-325-3p overexpression attenuated the degree of injury to the cardiac tissue, decreased the infarct sizes and downregulated the expression of the necrosis-related proteins RIPK1, RIPK3 and p-MLKL.

**Conclusions:**

The RIPK1/RIPK3/p-MLKL axis-induced necroptosis that occurred during MI was mediated by a miRNA module, miR-325-3p, which can effectively ameliorate the symptoms of MI by suppressing the expression of RIPK3.

## Background

Myocardial infarction is a leading cause of mortality worldwide and is usually caused by the pathological coronary artery occlusion [1, 2]. In China, according to the estimates from World Bank, more than 23 million will experience myocardial infarction annually by 2030 [3]. Risk factors, including smoking, metabolic syndrome and hypertension, could cause the higher morbidity and mortality seen in myocardial infarction cases [4]. The following mechanisms have been implicated in the progression of myocardial infarction: kallikrein-kinin [5], carbon metabolism [6], and renin–angiotensin–aldosterone system [4]. However, the key mechanism still needs further investigation.

In recent years, increasing evidence has suggested that programmed necrosis (also termed necroptosis, a novel mechanism of cell death that combines the features of necrosis and apoptosis) is closely related to various cardiovascular diseases (e.g., myocardial infarction and cardiac ischemia reperfusion injury) as a major contributor to cardiomyocyte cells death [7, 8]. In the process of necroptosis, the intracellular contents are released from the ruptured cells, which can trigger an innate immune response [9–11]. Previous studies have demonstrated that both RIPK1 (receptor interacting serine/threonine kinase 1) and RIPK3 (receptor interacting serine/threonine kinase 3) were involved in the regulation of necroptosis induced by H_2_O_2_ in cardiomyocytes [12]. Another study demonstrated that only RIPK3 overexpression, but not RIPK1 overexpression, was enough to induce the necroptosis of neonatal rat ventricular cardiomyocytes [13]. On the other hand, a deficiency of RIPK3 not only attenuated the death rate of cardiomyocytes in cardiac ischemia reperfusion injury mice [14], but also improved the symptoms of myocardial infarction and systemic inflammation induced by high-dose TNF-α or A20 deficiency [15].

MicroRNAs (miRNAs) are a class of small, but highly conserved, single-stranded non-coding RNA that are widely prevalent in plants, some viruses, humans and other animals. They engage in various physiological and developmental processes, such as cell division, apoptosis and the immune response [16]. In the past few years, many miRNAs have been reported to be involved in the progression of cardiovascular diseases, including atherosclerosis, heart failure and myocardial infarction [17]. Several frontier studies have reported the significance of some downregulated or upregulated miRNAs in the progression myocardial infarction [18–20]. However, to our best knowledge, few studies that have focused on the mechanism of miRNAs in myocardial infarction have been reported, particularly through the pathways involving RIPK1/RIPK3.

In this study, we aimed to identify more specific and efficient miRNAs that can ameliorate and improve the symptoms of myocardial infarction and can uncover the potential mechanism. We demonstrated that the upregulation of miR-325-3p largely restricted the progression and deterioration of MI in mice by suppressing the activation of RIPK3 and the subsequent necroptosis.

## Results

### Downregulation of miR-325-3p in MI mice

**Fig. 1:**
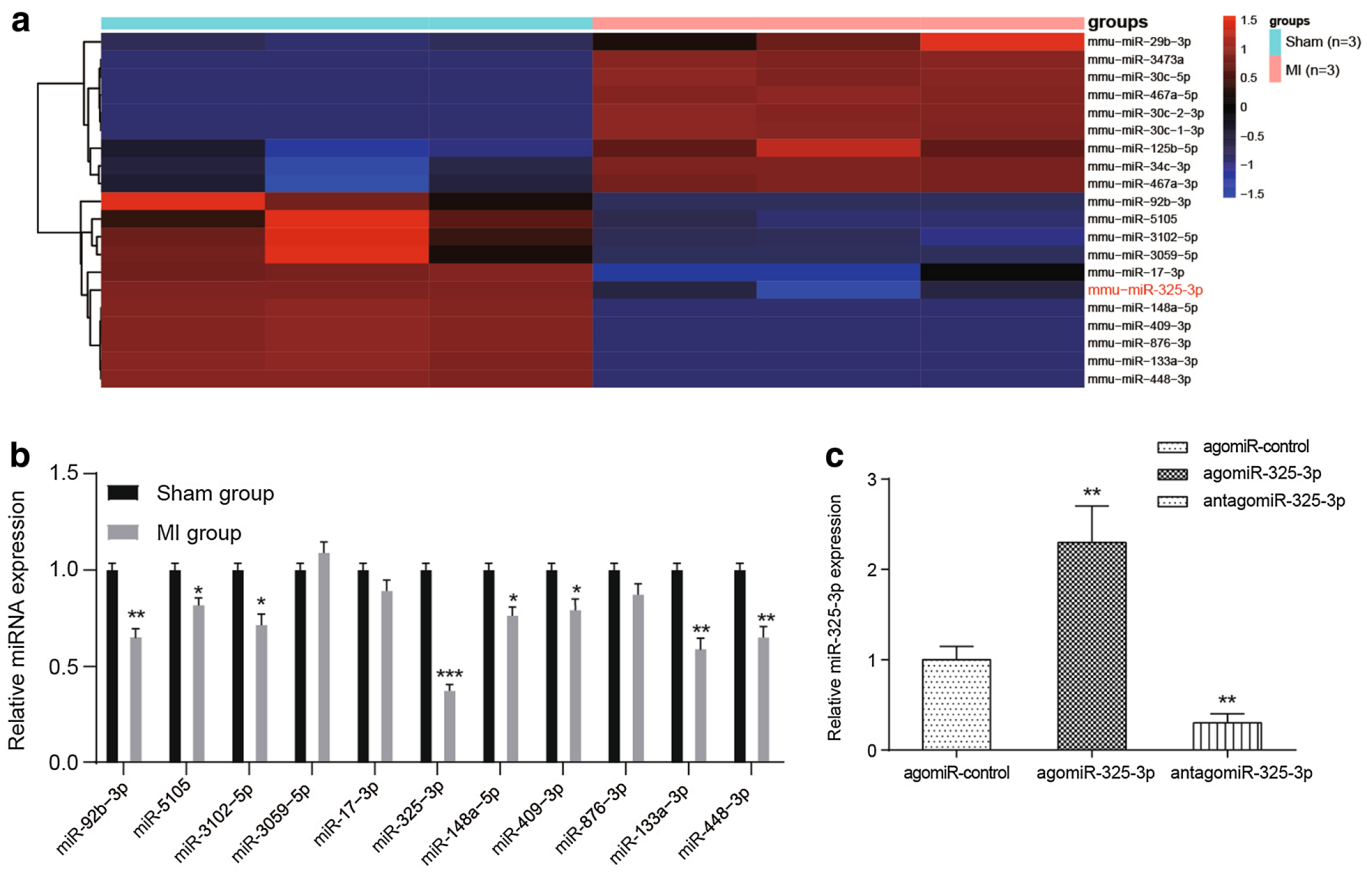
Expression features of miR-325-3p in MI mice. **a** The expression profiles of miRNAs in MI mice. Twenty miRNAs with the greatest changes in MI mice are shown. The miRNAs that have high expression or low expression are colored red and blue. **b** The expression of the downregulated miRNAs in MI tissues were analyzed by qRT-PCR analysis. *P < 0.05, **P < 0.01, ***P < 0.001 compared to the mice that received the sham operation. **c** The influence of agomiR-325-3p or antagomiR-325-3p on the expression of miR-325-3p in MI mice. **P < 0.01 compared to MI mice treated with agomiR-control. MI, myocardial infarction; agomiR-325-3p, miR-325-3p agomir; antagomiR-325-3p, miR-325-3p antagomir; agomiR-control, scrambled agomir or antagomir control

The differentially expressed miRNAs between sham-operated mice and MI mice were screened by miRCURYTM LNA Array v.18.0. MiRNAs with the most significant difference (n = 20) are shown in Fig. 1a, of which, 9 miRNAs were activated in MI mice, while the other 11 miRNAs were downregulated. The expression levels of the 11 downregulated miRNAs were further detected by qRT-PCR analysis. MiR-325-3p was found to have the most significant downregulation in MI tissues compared with that in other miRNAs (*P *< 0.001, Fig. 1b). The decreased expression of miR-325-3p in MI mice was further deteriorated with the administration of antagomiR-325-3p but was reversely upregulated with agomiR-325-3p treatment (*P *< 0.01, Fig. 1c).

### Correlation between miR-325-3p and serum myocardial enzymes in MI mice

**Fig. 2:**
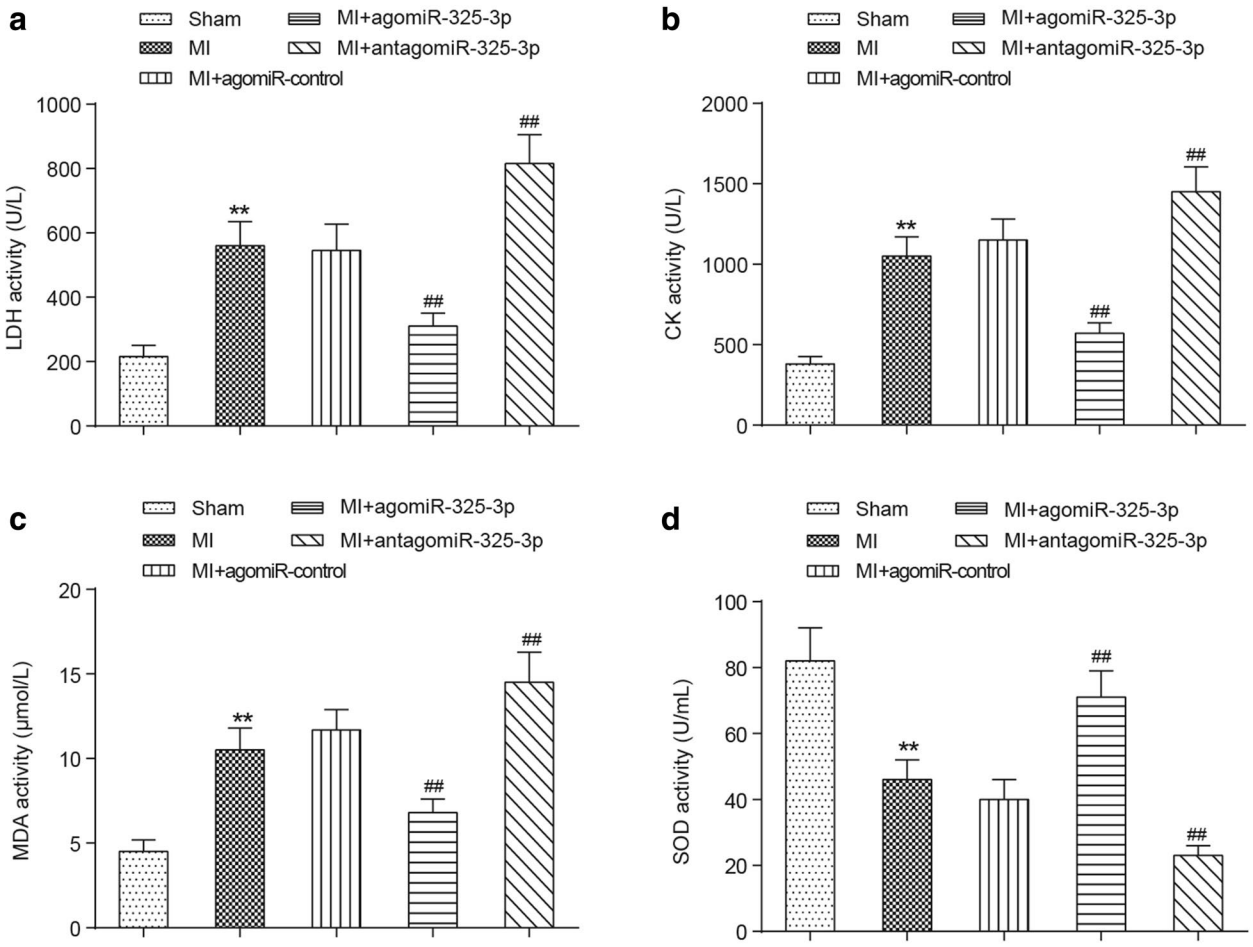
The change of myocardial enzyme activity in MI mice with miR-325-3p dysregulation. The adult male mice were randomly divided into the sham group, MI group, MI + agomiR-control group, MI + agomiR-325-3p group and MI + antagomiR-325-3p group. **a** The serum concentrations of LDH in different groups. **b** The serum concentration of CK in different groups. **c** The serum concentration of MDA in different groups. **d** The serum concentration of SOD in different groups. **P < 0.01 compared to the mice that received the sham operation, ^##^*P *< 0.01 compared to MI mice. MI, myocardial infarction; agomiR-325-3p, miR-325-3p agomir; antagomiR-325-3p, miR-325-3p antagomir; agomiR-control, scrambled agomir or antagomir control; LDH, lactate dehydrogenase; CK, phosphocreatine kinase; SOD, superoxide dismutase; MDA, malondialdehyde

Compared to the sham-operated mice, the serum levels of lactate dehydrogenase (LDH) (Fig. 2a), phosphocreatine kinase (CK) (Fig. 2b) and malondialdehyde (MDA) (Fig. 2c) were significantly increased in MI mice (*P *< 0.01), and they reached their highest level when the activity of miR-325-3p was suppressed by antagomiR-325-3p. The treatment of agomiR-325-3p obviously attenuated LDH, CK and MDA serum concentrations in MI mice (*P *< 0.01), although they are still slightly higher than the levels in normal mice. Conversely, the serum level of superoxide dismutase (SOD) was obviously reduced in MI mice and exhibited its lowest level in MI + antagomiR-325-3p-treated mice (*P *< 0.01), while miR-325-3p overexpression dramatically elevated the concentration of SOD in MI mice (*P *< 0.01) (Fig. 2d).

### Overexpression of miR-325-3p improves the cardiac function of MI mice

**Fig. 3:**
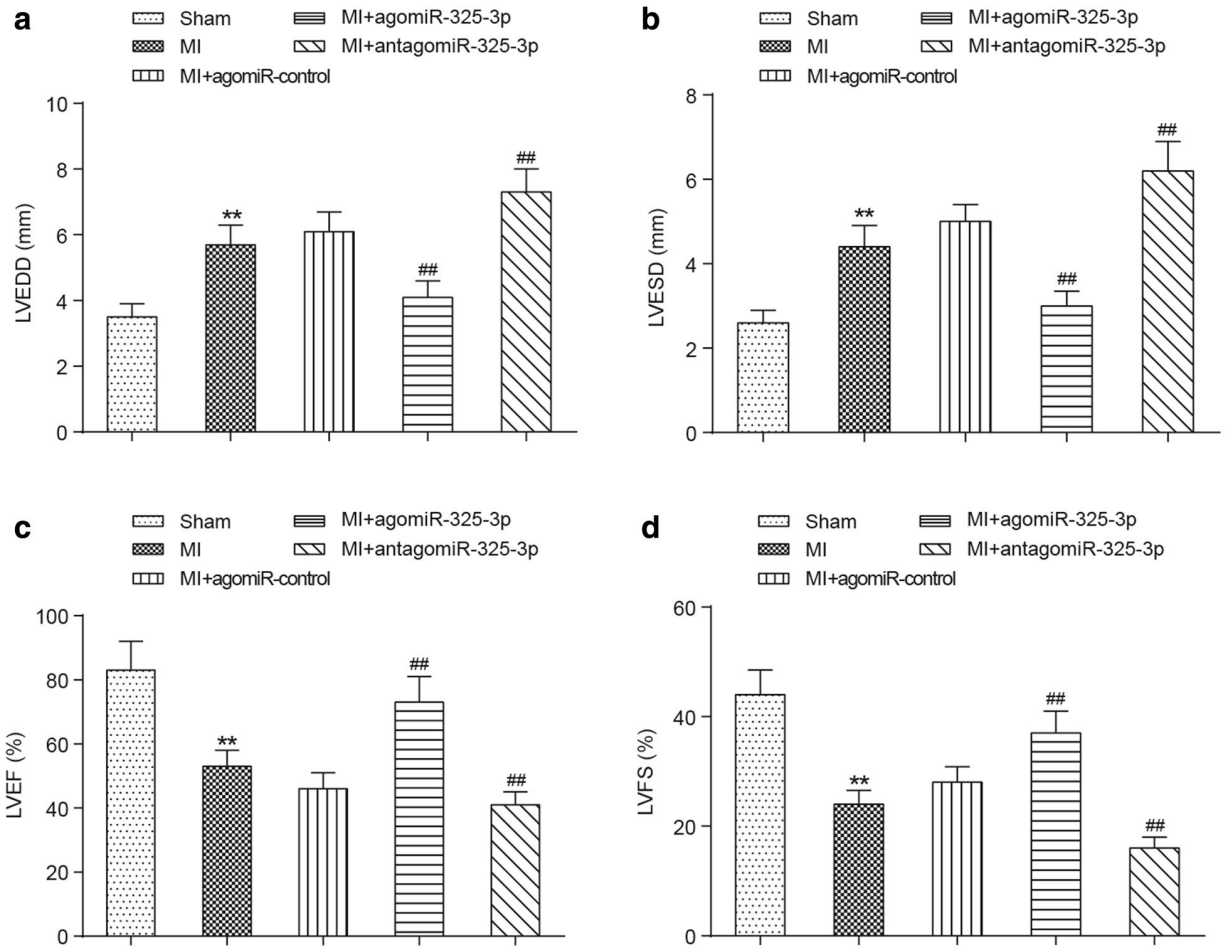
Influences of miR-325-3p dysregulation on the cardiac function parameters in MI mice. **a**, **b** The comparison of LVEDD and LVESD between the sham group, MI group, MI + agomiR-control group, MI + agomiR-325-3p group and MI + antagomiR-325-3p group mice. **c**, **d** The comparison of LVEF and LVFS between the sham group, MI group, MI + agomiR-control group, MI + agomiR-325-3p group and MI + antagomiR-325-3p group mice. **P < 0.01 compared to the mice that received the sham operation, ^##^*P *< 0.01 compared to MI mice. MI, myocardial infarction; agomiR-325-3p, miR-325-3p agomir; antagomiR-325-3p, miR-325-3p antagomir; agomiR-control, scrambled agomir or antagomir control; LVEDD, left ventricular end-diastolic diameter; LVESD, left ventricular end-systolic diameter; LVEF, left ventricular ejection fraction; LVES, left ventricular fractional shortening

The cardiac function of mice was assessed by echocardiography, as shown in Fig. 3. Both of the left ventricular end-diastolic diameter (LVEDD) and left ventricular end-systolic diameter (LVESD) were significantly increased in the MI mice (*P *< 0.01). AntagomiR-325-3p administration adversely caused the higher values of LVEDD and LVESD in MI mice (*P *< 0.01), while the agomiR-325-3p administration significantly attenuated the values of LVEDD and LVESD in MI mice (*P *< 0.01, Fig. 3a, b). What’s more, we calculated the values of left ventricular ejection fraction (LVEF) and left ventricular fractional shortening (LVES). The results showed that both LVEF and LVFS were decreased in MI mice (*P *< 0.01) and reached their lowest levels in antagomiR-325-3p-treated MI mice (*P *< 0.01). Conversely, LVEF and LVFS values in MI mice were upregulated and reached the near-normal level when treated with agomiR-325-3p (*P *< 0.01, Fig. 3c, d).

### Overexpression of miR-325-3p attenuates the cardiac damages in MI mice

**Fig. 4:**
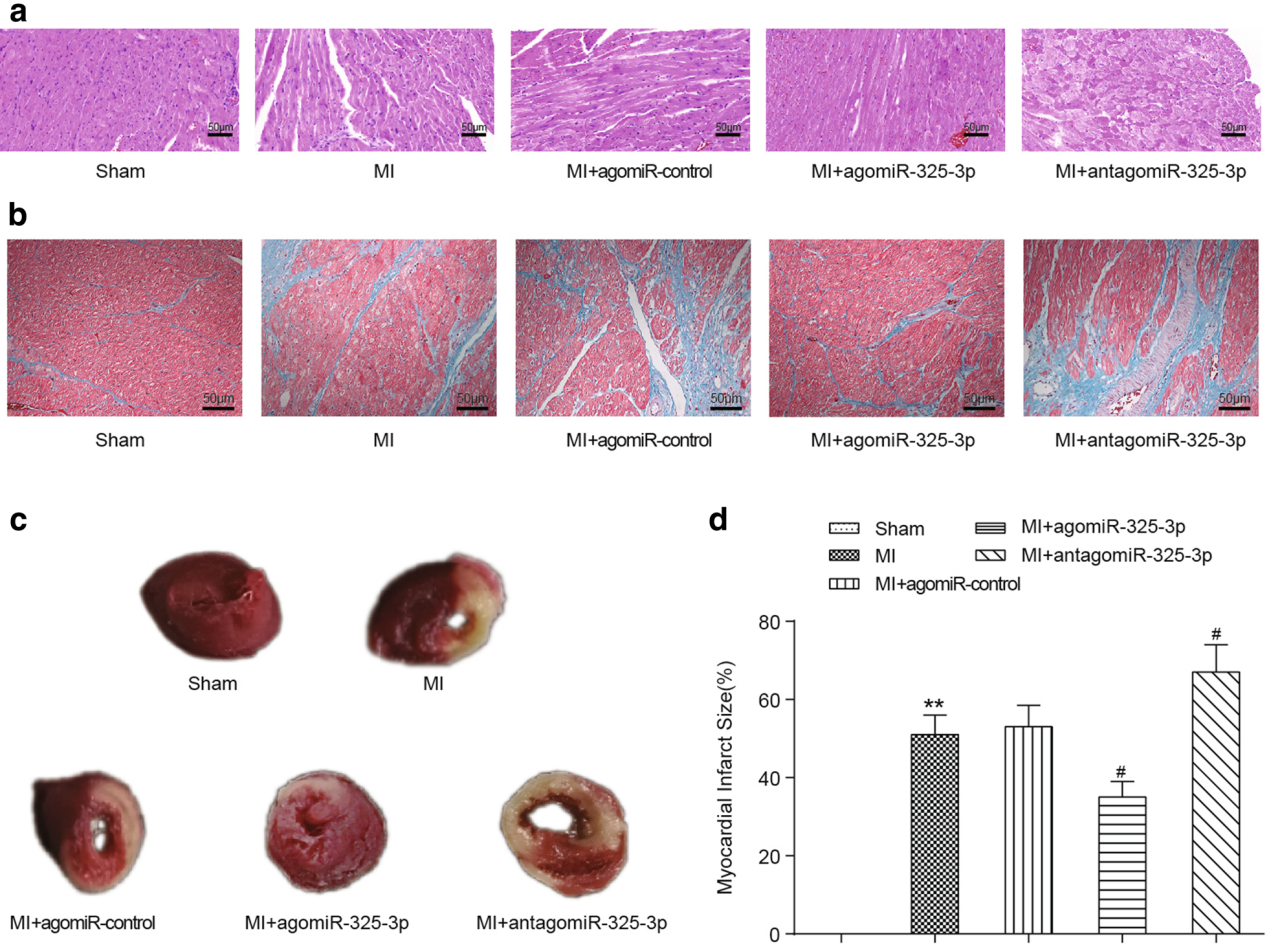
Pathological injury and the infarct size of cardiac tissues in MI mice with miR-325-3p dysregulation. **a** Representative images of cardiac tissues stained with H&E. **b** Representative images of cardiac tissues stained with Masson’s trichrome. The myocardial fibers and collagen are colored blue. **c** Representative images of cardiac tissues that were stained with TCC. **d** The comparison of infarct size among the groups. **P < 0.01 compared to the mice that received the sham operation, ^#^*P *< 0.05 compared to MI mice. MI, myocardial infarction; agomiR-325-3p, miR-325-3p agomir; antagomiR-325-3p, miR-325-3p antagomir; agomiR-control, scrambled agomir or antagomir control; H&E, hematoxylin and eosin; TCC, tetrazolium chloride

The pathological changes of heart tissue were assessed by H&E staining. As shown in Fig. 4a, the left ventricular areas of the mice that received the sham operation were clearly visible, with the myocardial fibers arranged neatly and no inflammatory cell infiltration in the myocardial interstitial space. However, in the heart tissue of MI mice, the boundary between the infarcted area and the non-infarcted area was clearly visible, a significantly increased infiltration of inflammatory cell was present, myocardial fibers in the infarcted area were dissolved and the myocardial stripes disappeared. In addition, compared to the mice that received the sham operation, the interstitial fibrosis and collagen accumulation in the cardiac tissues of MI mice were apparent (Fig. 4b). These phenomena were effectively improved by the administration of agomiR-325-3p (e.g., reduced interstitial fibrosis and collagen volume, decreased infiltration of inflammatory cells and normalized myocardial fibers) but were aggravated by the treatment of antagomiR-325-3p. TCC staining revealed that the size of myocardial infarction in MI mice increased to 50% and further increased to approximately 70% with the administration of antagomiR-325-3p. Conversely, agomiR-325-3p treatment caused the size of the myocardial infarction in MI mice to decrease to less than 40% (Fig. 4c, d).

### Overexpression of miR-325-3p inhibits myocardial tissue necroptosis in MI mice

**Fig. 5:**
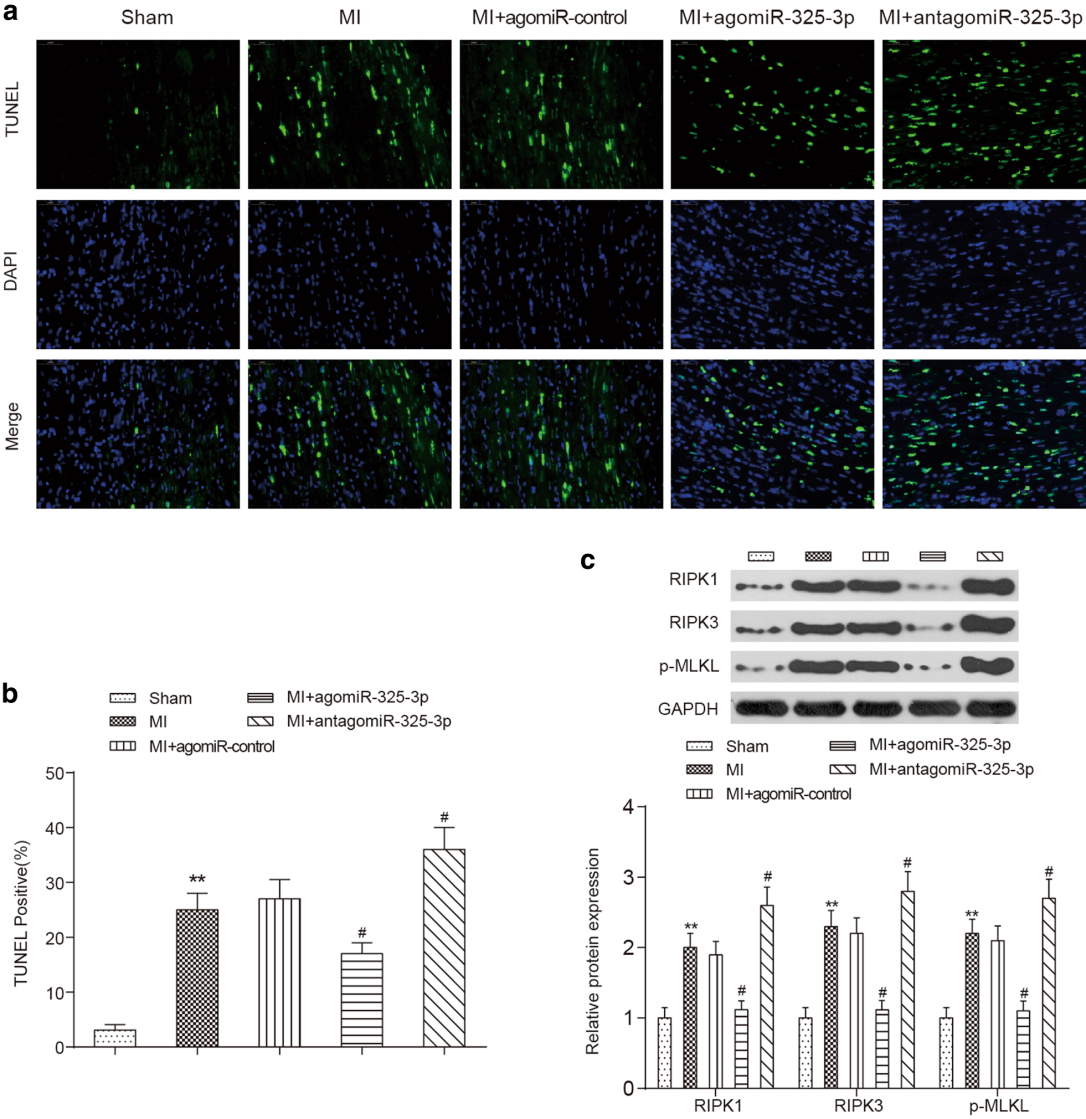
Influences of miR-325-3p dysregulation on the apoptosis of cardiomyocytes in MI mice. **a** Representative images of cardiac tissues that were stained with TUNEL (green), DAPI (blue) and the merge images of TUNEL and DAPI are shown. **b** The quantification of TUNEL-positive cells. **c** The Western blot results of RIPK1, RIPK3 and p-MLKL. Representative images (top) and quantitative comparison (bottom) are shown. **P < 0.01 compared to the mice that received the sham operation, ^#^*P *< 0.05 compared to MI mice. MI, myocardial infarction; agomiR-325-3p, miR-325-3p agomir; antagomiR-325-3p, miR-325-3p antagomir; agomiR-control, scrambled agomir or antagomir control; TUNEL, terminal deoxynucleotidyl transferase dUTP nick-end labeling; DAPI, 4-6-diamidino-2-phenylindole; RIPK1, receptor-interacting serine/threonine protein kinase 1; RIPK3, receptor-interacting serine/threonine protein kinase 3; p-MLKL, phosphorylated mixed-lineage kinase domain-like protein

The results of in situ TUNEL staining indicated that the necroptotic rate of cardiomyocytes in MI mice was significantly higher than that in the sham-operated mice (*P *< 0.01). The upregulation of miR-325-3p significantly reduced this rate in MI mice (*P *< 0.05), and the downregulation of miR-325-3p induced a higher necroptotic rate of cells in MI mice (*P *< 0.05) (Fig. 5a, b). We also detected the protein levels of necroptosis biomarkers, including RIPK1, RIPK3 and phosphorylated MLKL by Western blot. As shown in Fig. 5c, they were all obviously upregulated in MI mice compared to the levels in healthy mice (*P *< 0.01), and they reached their highest expression levels in the antagomiR-325-3p-treated MI mice. However, the administration of agomiR-325-3p greatly attenuated their expression levels in the cardiac tissues of MI mice (*P *< 0.05, Fig. 5c).

### MiR-325-3p suppress necroptosis cardiomyocytes under the hypoxia condition

**Fig. 6:**
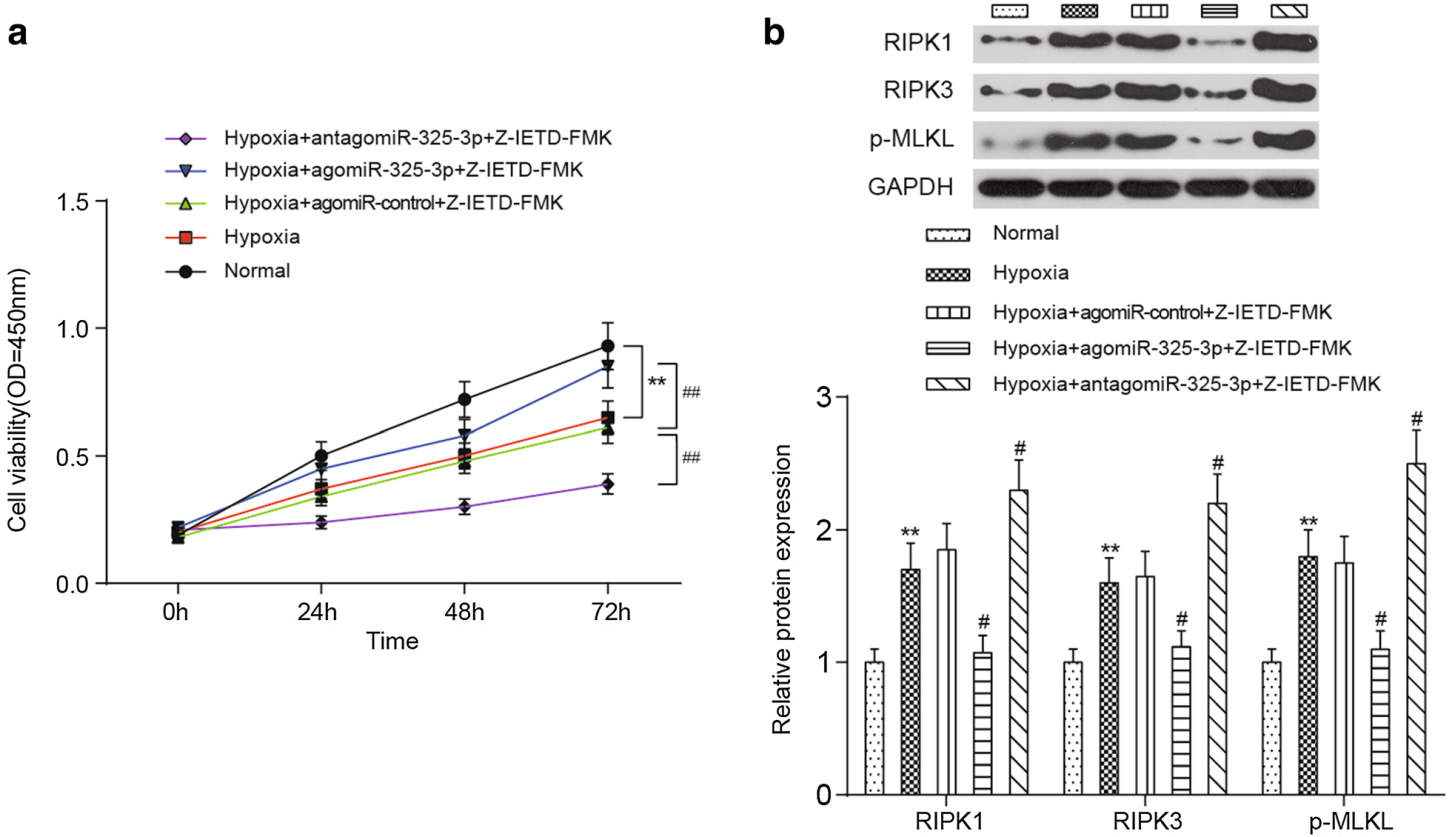
Influences of miR-325-3p dysregulation on the viability of cardiomyocytes in vitro. **a** Both the up- and downregulation of miR-325-3p affect the cell viability of hypoxia-induced cardiomyocytes. **b** The protein expression of RIPK1, RIPK3 and p-MLKL in cardiomyocytes is shown. Representative images (top) and quantitative comparisons (bottom) are shown. **P < 0.01 compared to cells cultured under normal conditions, ^##^*P *< 0.01 compared to cells treated with hypoxia + agomiR-control + Z-IETD-FMK. MI, myocardial infarction; agomiR-325-3p, miR-325-3p agomir; antagomiR-325-3p, miR-325-3p antagomir; agomiR-control, scrambled agomir or antagomir control; Z-IETD-FMK, a caspase inhibitor, benzyloxycarbonyl (Cbz)-Ile-Glu (OMe)-Thr-Asp (OMe)-FMK; RIPK1, receptor-interacting serine/threonine protein kinase 1; RIPK3, receptor-interacting serine/threonine protein kinase 3; p-MLKL, phosphorylated mixed-lineage kinase domain-like protein

The cardiomyocytes were isolated from the heart tissues of adult healthy mice, and then were cultured under hypoxic conditions (95% N_2_, 5% CO_2_). The viability of the cardiomyocytes was measured using a CCK8 kit. At 72 h, the viability of the cardiomyocytes cultured under hypoxic conditions were significantly lower than those cultured under normal conditions and they reached the lowest levels when antagomiR-325-3p was added into the medium along with the caspase-8 inhibitor E-IETD-FMK, which was used to eliminate the disturbance of apoptosis. In contrast, agomiR-325-3p effectively increased the viability of the cardiomyocytes cultured under hypoxic conditions, and this viability was close to the viability of the cardiomyocytes cultured under normal conditions (Fig. 6a). The results of the Western blot indicated that the expression levels of RIPK1, RIPK3 and p-MLKL were greatly upregulated in the hypoxia-induced cardiomyocytes (*P *< 0.01) and reached the highest levels when treated with antagomiR-325-3p. However, agomiR-325-3p administration caused the opposite consequences, which were a great reduction of the RIPK1, RIPK3 and p-MLKL expression levels in the hypoxia-induced cardiomyocytes (*P *< 0.05) (Fig. 6b). These results suggest that miR-325-3p enhance viability of the cardiomyocytes by suppressing necroptosis of the cardiomyocytes under the hypoxia condition.

### MiR-325-3p directly targets RIPK3

**Fig. 7:**
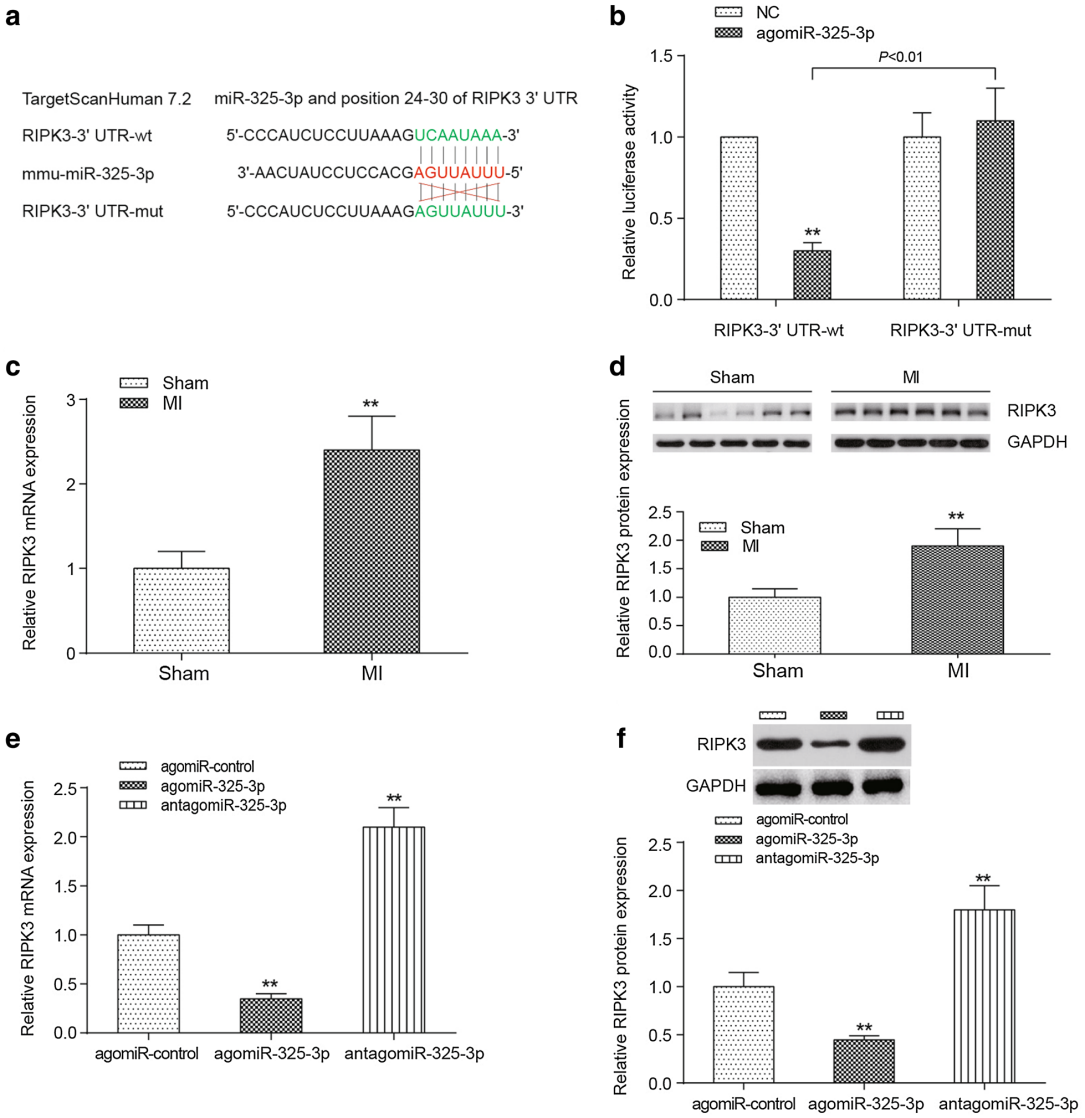
Target relationship between miR-325-3p and RIPK3. **a** The binding sites between the 3′UTR of RIPK3 and miR-325-3p predicted by TargetScanHuman 7.2. **b** A dual-luciferase reporter assay validated the target relationship between miR-325-3p and the 3′UTR of RIPK3. **P < 0.01 between the wild-type and mutated 3′UTR of RIPK3. **c**, **d** The differential expression of RIPK3 mRNA (**c**) or protein (**d**) in the sham-operated mice and the MI mice. **P < 0.01 compared to the mice that received the sham operation. **e**, **f** The influence of miR-325-3p dysregulation on the expression of RIPK3 mRNA (**e**) and protein (**f**) in MI mice. **P < 0.01 compared to MI mice treated with agomiR-control. MI, myocardial infarction; agomiR-325-3p, miR-325-3p agomir; antagomiR-325-3p, miR-325-3p antagomir; agomiR-control, scrambled agomir or antagomir control; RIPK3, receptor-interacting serine/threonine protein kinase 3

MiR-325-3p was predicted to possess the potential targeting ability to the 3′UTR of *RIPK3* (at the position of 24–30), using an online application (http://www.targetSCAN.org) (Fig. 7a). A dual-luciferase reporter assay was performed to verify the interaction between miR-325-3p and *RIPK3*. The results demonstrated that the upregulation of miR-325-3p could significantly suppress the relative luciferase activity of firefly in the RIPK3-wt group (*P *< 0.01), but failed to affect the fluorescence intensity of firefly in the RIPK3-mut group (*P *> 0.05, Fig. 7b). Real-time RT-PCR and Western blots were performed to verify the mRNA and protein expression levels of RIPK3, respectively, in the heart tissues of MI mice compared to those in sham mice, and both expression levels were increased significantly (Fig. 7c, d, *P *< 0.01). The activation of miR-325-3p (induced by agomir) significantly attenuated the expression of RIPK3 mRNA and protein expression in MI mice. Downregulation of miR-325-3p by antagomir caused a higher expression level of RIPK3 in MI mice (Fig. 7e, f, *P *< 0.01).

### Silencing of RIPK3 increases the viability of cardiomyocytes under hypoxic conditions

**Fig. 8:**
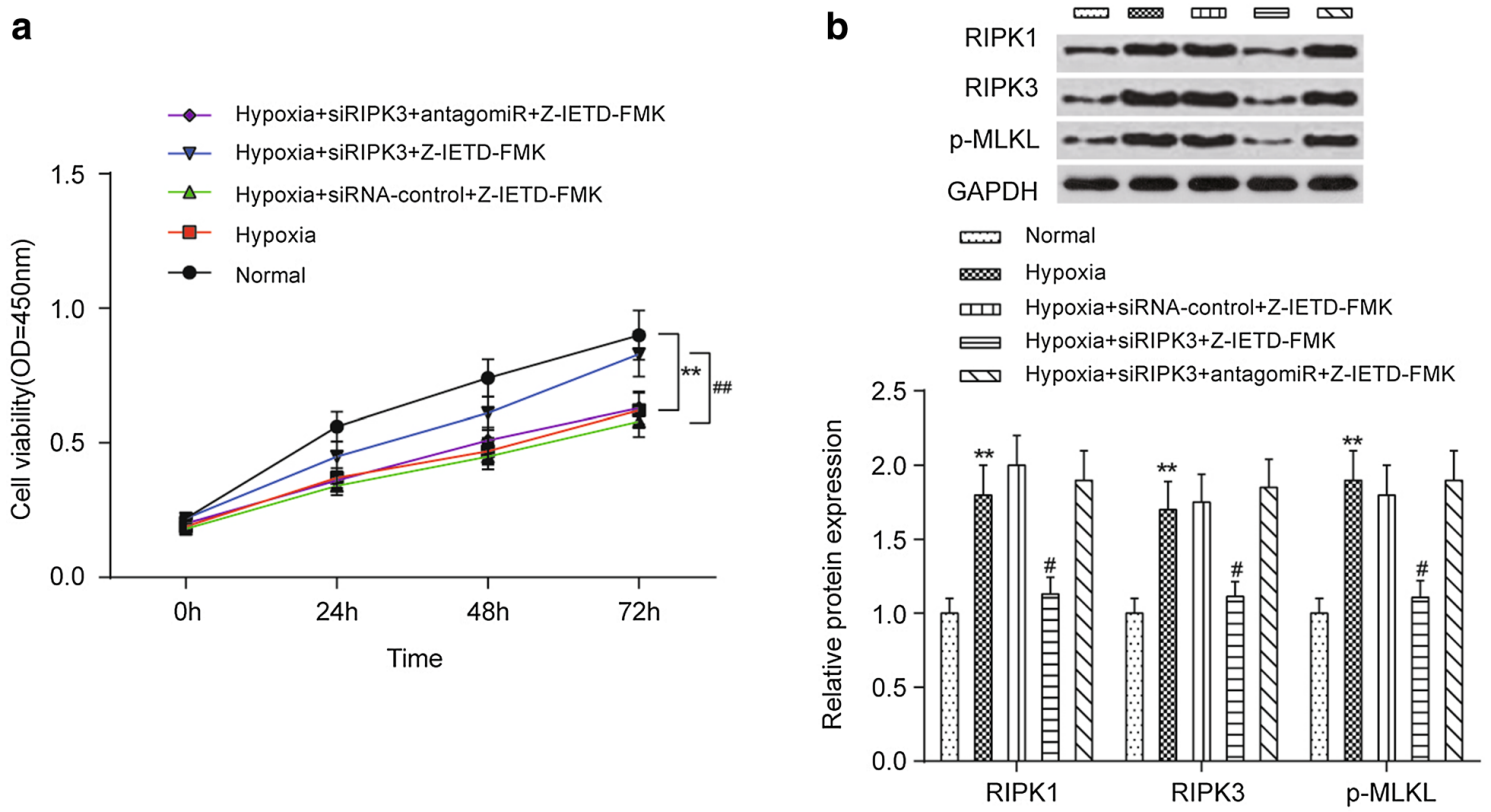
Influences of RIPK3 dysregulation on the viability of cardiomyocytes in vitro. **a** RIPK3-targeting siRNA increased the cell viability of cardiomyocytes under hypoxic conditions. **b** The protein expression of RIPK1, RIPK3 and p-MLKL in cardiomyocytes affected by RIPK3-targeting siRNA. Representative images (top) and quantitative comparison (bottom) are shown. **P < 0.01 compared to cells cultured under normal conditions, ^#^*P *< 0.05 and ^##^*P *< 0.01 compared to cells treated with hypoxia + siRNA-control + Z-IETD-FMK. MI, myocardial infarction; antagomiR-325-3p, miR-325-3p antagomir; siRIPK3, a RIPK3-targeting small interfering RNA; siRNA-control, scrambled siRNA control; Z-IETD-FMK, a caspase inhibitor, benzyloxycarbonyl (Cbz)-Ile-Glu (OMe)-Thr-Asp (OMe)-FMK; RIPK1, receptor-interacting serine/threonine protein kinase 1; RIPK3, receptor-interacting serine/threonine protein kinase 3; p-MLKL, phosphorylated mixed-lineage kinase domain-like protein

The protection effect of RIPK3 silencing on the viability of cardiomyocytes was evaluated in vitro (Fig. 8). After 72 h of RIPK3 silencing, compared with the viability of cardiomyocytes cultured under normal conditions, the viability of cells cultured under hypoxic conditions was significantly decreased (*P *< 0.01), but this decline was significantly alleviated when the hypoxia-induced cardiomyocytes were pre-transfected with RIPK3-targeted siRNA (*P *< 0.01); this effect could be eliminated by use of antagomiR-325-3p (Fig. 8a). Western blots indicated that the upregulated expression levels of RIPK1, RIPK3 and p-MLKL in the hypoxia-induced cardiomyocytes were significantly attenuated when the cells were pre-transfected with RIPK3-targeted siRNA (*P *< 0.05, Fig. 8b).

## Discussion

Necroptosis is a caspase-independent variant of regulated cell death that critically depends on the activity of RIPK3 and p-MLKL [21]. As a crucial, lethal signaling pathway, necroptosis engages in the development of various diseases, including ischemic injury, viral infection, hepatic disorders and many others [22]. Particularly in recent years, the role of necroptosis in the pathogenesis of cardiovascular pathologies has attracted huge attention from researchers, and several studies have indicated that suppressing the activity of necrosis significantly improved the following: the symptoms of myocardial hypertrophy [23], heart failure triggered by ischemia/reperfusion [24], and even myocardial infarction [13]. In this study, we established the mouse model of myocardial infarction and found that the apoptotic rate of cardiomyocytes in MI mice was significantly higher than that in healthy mice. This was accompanied by the changed expression profiles of miRNAs, pathologically changed heart tissue, impaired cardiac function and dysfunctional myocardial enzymes.

By a miRNA microarray analysis, many miRNAs, including miR-29b-3p, miR-3473a, miR-30c-5p, miR-3102-5p, miR-448-3p, and miR-325-3p, were revealed to be evidently changed in the heart tissues of MI mice. MiRNAs are a class of single-stranded non-coding RNAs, and their overexpression, deletion, epigenetic modification or polymorphism significantly influences the expression of their target mRNAs at the posttranscriptional level [25]. Recent evidence has shown that the dysfunction of miRNAs is closely associated with the progression and prognosis of cardiac disorders, such as cardiomyocyte hypertrophy, myocardial infarction and heart failure [26]. For example, the cardiomyocyte-specific overexpression of miRNA-30c caused severely dilated cardiomyopathy in mice [27], Decreased plasma levels of miR-145 have also been found to be associated with acute myocardial infarction [28]. Additionally, it has been reported that the expression levels of miR-103/107, miR-2861, and miR-874 are significantly upregulated in MI heart tissue, and thus, the elevated expression levels promote the death of cardiomyocytes. On the contrary, miR-155 and miR-873 are downregulated, and the consequence was the inhibition of cardiomyocyte death through the RIPK1/RIPK3-based necroptosis pathway. In the list of differentially expressed miRNAs in MI mice, miR-133a was found to be significantly downregulated in the heart tissues of myocardial infarctions by Bostjancic et al., and this was consistent with our results [29]. MiR-448 was also implicated in myocardial infarction through using a combined microarray analysis of miRNA and mRNA in a study reported by Wang et al. [30]. The results of downregulated miR-325-3p were controversial, previous results indicate that the overexpression of miR-325 in cardiomyocytes greatly enlarged the sizes of myocardial infarctions through the suppression of apoptosis repressor with caspase recruit domain (ARC) [31]. Thereby, whether the downregulation of miR-325-3p played a role in myocardial infarction has been investigated, and the results suggest that the inhibited expression of miR-325-3p, which was induced by a specific antagomir, closely associated with the larger myocardial infarct size, higher rate of cardiomyocyte apoptosis, worse cardiac function and myocardial enzyme activities in MI mice; conversely, these symptoms were significantly relieved by the administration of agomiR-325-3p, an optimized alternative to miRNA-325-3p mimics that has the ability to induce the stable upregulation of endogenous miRNA.

Although the expression levels of RIPK1, RIPK3 and phosphorylated MLKL were increased in the heart tissue of MI mice and hypoxia-induced cardiomyocytes, only the knockdown of RIPK3 by a specific siRNA could significantly increase the viability of cardiomyocytes cultured under hypoxic conditions to the viability of cells cultured under normal conditions. Additionally, bioinformatics analysis and a dual-luciferase assay revealed that RIPK3 is targeted by miR-325-3p. The expression level of RIPK3 protein was exactly decreased in the agomiR-325-3p-treated MI mice compared to the level in MI mice, and this was accompanied by the lower expression of phosphorylated MLKL, reduced myocardial infarct size, reduced apoptotic rate of cardiomyocytes, and improved cardiac function and myocardial enzyme activities. As mentioned above, both the activation of RIPK3 and the subsequent phosphorylation of MLKL are crucial steps for the initiation of necroptosis; however, the phosphorylation of MLKL strictly depends on the activity of RIPK3 [10]. On the other hand, RIPK1 has been reported to not be the sole activator of RIPK3 in murine and human cells; at least two other proteins can physically interact with RIPK3 [32–34]. The importance of RIPK3 in necroptosis has also been confirmed by several previous studies based on the RIPK3^−/−^ animal model. For example, the knockout of RIPK3 endowed the mice with the ability to resist heart failure that was triggered by ischemia/reperfusion, and this was independent of the activation of RIPK1 or MLKL [13, 35]. In addition, mice deficient for RIPK3 (RIPK3^−/−^) presented a significant improvement in ejection fraction, hypertrophy and inflammatory cells infiltration in the heart tissues of MI rats [13]. In line with these notions, the dysregulation of RIPK3 may play an indispensable role in the development of necroptosis-associated diseases, which are presented as myocardial infarction, and RIPK3 may be a candidate target for new drug designs against these diseases.

However, limitations still exist in this study. The dysregulation of miRNAs in MI mice consisted of changes in 20 miRNAs, but our present study reports only how the upregulation of miR-325-3p improved the symptoms of myocardial infarction, but the roles of the remaining miRNAs involved in myocardial infarction still remain unknown. These questions will be the central focus of our future studies.

## Conclusions

In conclusion, our present findings indicate that the dysregulation of miRNAs was obviously present in the MI mice. Our findings also indicate that miR-325-3p overexpression, induced by a specific agomiR, evidently alleviated these MI-associated symptoms via the suppression of the RIPK3-based necroptosis pathway.

## Methods

### Experimental animals and treatments

In this study, all animal-based experimental procedures were strictly performed following the instructions of the Animal Experimental Center of the Affiliated Huaian No.1 People’s Hospital of Nanjing Medical University. Adult, male, wild-type (WT) C57BL/6J mice weighing 20–25 g were purchased from SLAC Laboratory Animal Co., Ltd. (Shanghai, China) and were individually kept in a specific, pathogen-free and temperature-controlled room (25 °C ± 1 °C) with the humidity set at 60–80% and 12/12 h circadian rhythm. Before starting the experiments, all mice were acclimated to their new environment for 1 week. During the experiments, standard laboratory food (Dashuo Laboratory Animal Technology Co., Chengdu, China) and water were available ad libitum, but fasting for 12 h was carried out prior to starting the experiments.

### Establishment of the myocardial infarction mouse model

The MI mouse model was established by coronary artery ligation. Briefly, the mice were anesthetized with inhaled ether for 3 min. Then, to expose the heart, the left thoracotomy was performed at the left, fourth intercostal space with the support of mechanical ventilation generated by a small animal ventilator (model SN-480-7 Shinano, Tokyo, Japan). After opening the pericardium, the left anterior descending coronary artery below or 2 mm distal to the left atrial appendage was immediately ligated by a 5-0 Prolene suture (Ethicon, Inc., Somerville, N.J.). A 2-0 suture was used to stitch the thorax and the skin. Similar surgical processes (except for the ligation) were performed on the mice from the sham group. All operations were performed under sterile conditions.

### RNA isolation, miRNA microarray and real-time RT-PCR

The total RNA was extracted from the heart tissue and cells and was performed as previously described [36]. A miRNA microarray analysis was performed with the miRCURY LNA™ microRNA Array (v.18.0) by KangChen-Biotech (Shanghai, China). The total miRNAs were extracted for chip hybridization. The expression levels of miRNAs were represented by the fluorescence intensity values. The differential expression criteria were set as fold change > 2, *P *< 0.05. For the RT-PCR analysis, the cDNA was reverse transcribed from the total RNA (2.0 μg) using the Quantscript RT Kit (Tiangen-Biotech, Beijing China). For the miRNA analysis, real-time RT-PCR was performed using the TanMan^®^ microRNA Assay Kit (Life Technologies, Carlsbad, CA. USA); for the mRNA analysis, iQ SYBR Green Supermix (Bio-Rad Laboratories, Hercules, CA, USA) was used. The data analysis was performed using the 2^−ΔΔCt^ method with the internal control of U6 (for miRNA quantification) and GAPDH (for mRNA quantification). The primer sequences for real-time RT-PCR are shown in Table 1.

**Table 1:** Primers for qRT-PCR

Gene	Sequence (5′–3′)
miR-92b-3p	
Forward primer	CCGCCCTGCTCACGTTAT
Reverse primer	AGTCTCAGGGTCCGAGGTATTC
miR-5105	
Forward primer	GGCGCCGCTCGT
Reverse primer	CAGTGCGTGTCGTGGAGT
miR-3102-5p	
Forward primer	GTGGTGCAGGCAGGA
Reverse primer	CAGTGCGTGTCGTGGAGT
miR-3059-5p	
Forward primer	TTCCTCTCTGCCCATAG
Reverse primer	CAGTGCGTGTCGTGGAGT
miR-17-3p	
Forward primer	CAGTAAAGGTAAGGAGAGCTCAATCTG
Reverse primer	CATACAACCACTAAGCTAAAGAATAATCTGA
miR-148a-5p	
Forward primer	TCAGTGCACTACAGAACTTTGT
Reverse primer	GCTGTCAACGATACGCTACGT
miR-409-3p	
Forward primer	AGGAATGTTGCTCGGTGA
Reverse primer	CAGTGCGTGTCGTGGAGT
miR-876-3p	
Forward primer	TGGATTTCTTTGTGAATCACCA
Reverse primer	GTGATTCACAAAGAAATCCATT
miR-133a-3p	
Forward primer	TTTGGTCCCCTTCAACCAGCTG
Reverse primer	TAAACCAAGGTAAAATGGTCGA
miR-448-3p	
Forward primer	GCTGAGGGAGATATCGGCGCC
Reverse primer	GGAACACGCATGGCAGATCC
miR-325-3p	
Forward primer	GCCAGCACCTTCACAAAGTAGC
Reverse primer	CCATGCTAGACAACAGCTCTG
RIPK3	
Forward primer	CCTCTCAGTCCACACTCCGA
Reverse primer	TGACGCACCAGTAGGCCAT
GAPDH	
Forward primer	AGCCACATCGCTCAGACAC
Reverse primer	GCCCAATACGACCAAATCC
U6	
Forward primer	ATTGGAACGATACAGAGAAGATT
Reverse primer	GGAACGCTTCACGAATTTG

### Animal transfection in vivo

AgomiR-control, agomiR-325-3p and antagomiR-325-3p were synthesized by BGI (Shenzhen, China). AgomiR-325-3p is the optimized version of a miR-325-3p mimic, and its in vivo delivery resulted in the same effects of the upregulation of endogenous miR-325-3p; agomiR-control was designed with a minimum homology to miRNA, and its delivery should not cause any influence to the expression of endogenous proteins. However, antagomiRNA-325-3p is the complementary single-stranded RNA analog of miRNA-325-3p that could specifically and efficiently inhibit the activity of endogenous miR-325-3p. At the end of the development of an MI model, 0.1 mM of either agomiR-325-3p, agomiR-control or antagomiR-325-3p (prepared by sterile phosphate-buffered saline) was microinjected into mouse hearts from the correct MI group. Based on this selection, these mice were further divided into the MI group (receiving 10 μL sterile PBS), MI + agomiR-325-3p group (receiving 10 μL agomiR-325-3p), MI + antagomiR-325-3p group (receiving 10 μL antagomiR-325-3p) and MI + agomiR-control group (receiving 10 μL agomiR-control).

### Assessment of cardiac function and myocardial infarct size

For a cardiac functional assessment, the mice were anesthetized with isoflurane and the following processes were performed as previously described [37]: the values of LVEDD, LVESD, LVEF and LVES were measured using the Sequoia 512 echocardiography system with a 15-MHz linear transducer (Siemens, Erlangen, Germany). In addition, the size of the myocardial infarction was measured by direct triphenyl tetrazolium chloride (TCC) staining according to the procedures described by Lie, et al. [38]. In brief, the hearts were excised from the sacrificed mice, washed with ice-cold PBS, frozen at − 20 °C for 30 min, and then transversely cut across the left ventricles. The slices (5 per heart, approximately 3 mm thick) were incubated in 1% TTC solution (pH = 7.4, Beyotime-Biotech, Beijing, China) at 37 °C for 30 min. The viable areas were stained red, but the infarcted areas cannot be stained, and thus, appeared pale white. Then, the sections were photographed and the infarct sizes were calculated by computerized planimetry using ImageJ v1.8.0 (National Institutes of Health, Bethesda, USA).

### Myocardial enzyme determination

Seventy-two hours after transfection, the blood from the inner canthus vein was collected and kept at 4 °C for 4 h, and the serum was separated by centrifugation and was stored at a temperature of − 80 °C. The serum concentrations of LDH, CK, SOD and MDA were measured using commercial kits (Sangon-Biotech, Shanghai, China; Solarbio, Beijing, China).

### Histopathological examination and TUNEL staining

Following anesthesia, the heart tissues were immediately excised and placed in a 4% paraformaldehyde solution. This was followed by dehydration, cleaning, and paraffin embedding. The paraffin blocks were cut into 4 μm sections using a microtome (Leica M650; Leica Microsystems GmbH, Wetzlar, Germany), and the sections were stained with hematoxylin and eosin (H&E, Solarbio) for histopathology and Masson’s trichrome (Sigma-Aldrich, St. Louis, MO, USA) for fibrosis. The histology of the myocardium was examined with light microscopy (Olympus, Tokyo, Japan).

The heart sections were also used to assess myocardial cell apoptosis through an in situ terminal deoxynucleotidyl transferase-mediated dUTP nick end-labeling (TUNEL) assay (Roche Diagnostics, Mannheim, Germany). In brief, following the routine deparaffinization and rehydration, antigen retrieval was performed on the sections (4 μm) by boiling in sodium citrate solution for 2 min. After washing with PBS three times, TUNEL staining was performed at 37 °C for 1 h. After washing again, these sections were stained with 4′,6-diamidino-2-phenylindole (DAPI) (Invitrogen, CA, USA). After washing another three times, the sections were photographed with a fluorescent microscope and the number of cells was counted using the pre-installed image analysis system (Olympus, Tokyo, Japan). The myocardial apoptosis ratio was taken as the apoptotic cell number (green)/the total cell number (blue) × 100%.

### Isolation of cardiomyocytes from mice and the induction of hypoxia

Cardiomyocytes were isolated from adult male C57BL/6J mice (approximately 10 weeks old) by proteolytic digestion and differential plating and were cultured in the DMEM/F12 medium supplemented with 10% fetal bovine serum (Gibco, Grand Island, NY, USA), 10 µg/mL holo-transferrin, 10 µg/mL insulin, 100 µM 5-bromodeoxyuridine, 1% ampicillin-streptomycin in a humidified incubator with 5% CO_2_. The cells were divided into five groups based on the following treatment conditions: cultured at normal conditions (normal group); cultured at hypoxic conditions (95% N_2_ and 5% CO_2_) (hypoxia group); cultured at hypoxic conditions and simultaneously treated with agomiR-control and the caspase inhibitor Z-IETD-FMK (hypoxia + agomiR-control + Z-IETD-FMK group); cultured at hypoxic conditions and treated with agomiR-325-3p and Z-IETD-FMK (hypoxia + agomiR-325-3p + Z-IETD-FMK group); and cultured at hypoxic conditions and treated with antagomiR-325-3p and Z-IETD-FMK (hypoxia + antagomiR-325-3p + Z-IETD-FMK group). The cell viability was detected by a CCK8 kit (Dojindo, Kumamoto, Japan), the optical density was measured by a microplate spectrophotometer at 450 nm (Wellscan MK3, Thermo/Labsystems, Finland).

Then, the cell-based assay was performed again with the following changes: agomiR-325-3p was replaced with the RIPK3-targeting siRNA (hypoxia + siRIPK3 + Z-IETD-FMK group) and agomiR-control was replaced with siRNA-control (hypoxia + siRNA-control + Z-IETD-FMK group), to investigate the effects of RIPK3 silencing on the viability of cardiomyocytes in vitro. The following sequences were referenced from the previous report by Zhao, et al. [23]: siRIPK3, 5′-GCG GGG UCA GGA UCG AGA GAU U dUdU-3′ and siNC, 5′-GUG CGU UGC UAG UAC CA AC dUdU-3′. At the same time, the cells in the hypoxia + siRIPK3 + Z-IETD-FMK group were cotreated with antagomiR-325-3p (i.e., the hypoxia + siRIPK3 + antagomiR-325-3p + Z-IETD-FMK group).

### Western blot assay

Lysates of heart tissue and cultured cells were prepared in RIPA buffer (Solarbio) supplemented with a protease inhibitor cocktail (Abmole, Houston, USA). The total protein content (80 μg) was segregated on 12% SDS–polyacrylamide gels and was then transferred onto 0.45 μm nitrocellulose membranes (Millipore, Plano, TX, USA). After being blocked in PBS-T buffer containing 5% fat-free milk at 37 °C for 2 h, the membranes were incubated with rabbit anti-RIPK1 (1:1200, Cell Signaling, Danvers, USA), anti-RIPK3 (1:2000, ProSci, San Diego, USA), anti-p-MLKL (1:2000, Abcam, Cambridge, MA, USA) or anti-GAPDH (1:2000, Abcam) antibodies at 4 °C overnight. After incubation with the horseradish peroxidase-conjugated secondary antibodies, the protein bands were visualized using an ECL kit (Sigma, USA).

### Dual-luciferase reporter gene assay

The targets of miR-325-3p were predicted by TargetScanHuman v7.2 (http://www.targetSCAN.org), and RIPK3 was considered to be the best candidate. To verify the predicted relationship between miRNA-325-3p and the 3′UTR of RIPK3, a dual-luciferase reporter assay was performed. Firefly luciferase reporter vectors encoding wild-type (wt) and the specifically mutated (mut) 3′UTR of RIPK3 (GV272-RIPK3) were commercially obtained from GeneChem (Shanghai, China). The HEK293T cells were cultured in DMEM/high glucose medium supplemented with 10% FBS. For the luciferase assay, the HEK293T cells (1 × 10^5^) were cotransfected with the recombinant plasmid GV272-RIPK3 and the *Renilla*-encoding vector pRL-SV50 (Promega, Madison, WI, USA), using Lipofectamine 2000 (Invitrogen). In addition, agomiR-325-3p or antagomiR-325-3p was added into the medium. The luciferase activities were measured using the dual-luciferase reporter assay system (Promega, USA), and the results were normalized to the *Renilla* luciferase activity.

### Statistical analysis

Every experiment was performed at least three times. The statistical analysis and the graph manipulation were conducted using GraphPad Prism 6.0 software. The data are presented as the mean ± standard deviation (SD). Student’s t-test was utilized to evaluate the difference between two groups, while a comparison among multiple groups was conducted by one-way ANOVA. A two-tailed *P*-value ≤ 0.05 was considered to be statistically significant.

## Data Availability

The datasets used and analysed during the current study are available from the corresponding author on reasonable request.

## Abbreviations

LDH: lactate dehydrogenase
LVESD: left ventricular end-systolic diameter
MI: myocardial infarction
miRNAs: microRNAs
p-MLKL: phosphorylated MLKL
SOD: superoxide dismutase
TCC: triphenyl tetrazolium chloride
WT: wild-type
