# Correction to: MicroRNA-325-3p protects the heart after myocardial infarction by inhibiting RIPK3 and programmed necrosis in mice

**DOI:** 10.1186/s12867-019-0136-9

**Published:** 2019-08-06

**Authors:** Dong-Ying Zhang, Bing-Jian Wang, Min Ma, Kun Yu, Qing Zhang, Xi-Wen Zhang

**Affiliations:** 1 Department of Cardiology, The Affiliated Huaian No.1 People’s Hospital of Nanjing Medical University, No.1 West Huanghe Road, Huaiyin District, Huaian, 223300 Jiangsu China; 2 Department of Cardiology, The Sixth People’s Hospital of Chengdu, Chengdu, 610051 China

## Correction to: BMC Molecular Biol (2019) 20:17 10.1186/s12867-019-0133-z

The original article [1] contains an error whereby Fig. 7 displays incorrect results; the correct version of Fig. 7 can be viewed ahead in this Correction article and should be considered in place of the original article’s version of Fig. 7.

**Fig. 7:**
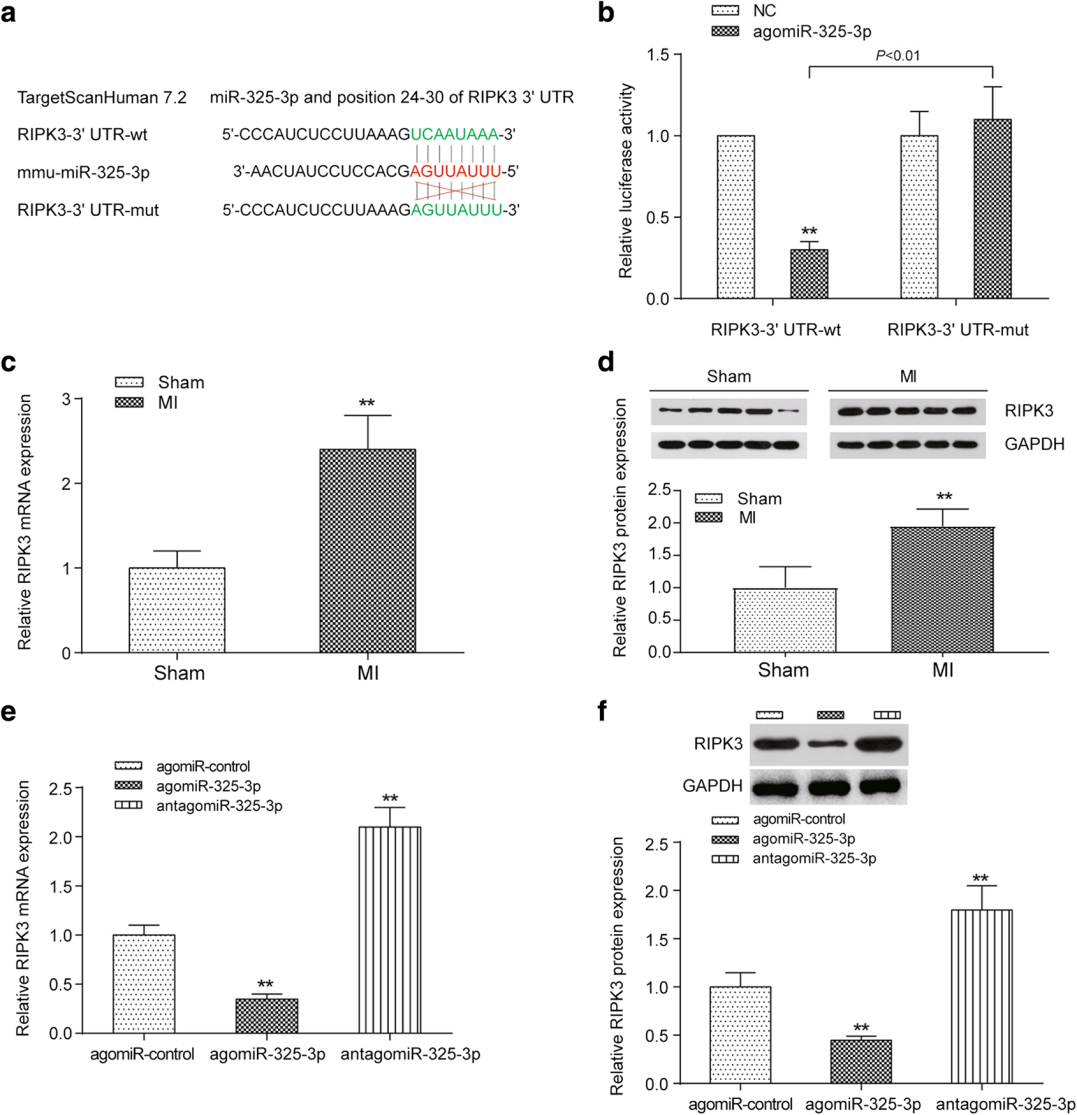
Target relationship between miR-325-3p and RIPK3. **a** The binding sites between the 3′UTR of RIPK3 and miR-325-3p predicted by TargetScanHuman 7.2. **b** A dual-luciferase reporter assay validated the target relationship between miR-325-3p and the 3′UTR of RIPK3. **P < 0.01 between the wild-type and mutated 3′UTR of RIPK3. **c**, **d** The differential expression of RIPK3 mRNA (**c**) or protein (**d**) in the sham-operated mice and the MI mice. **P < 0.01 compared to the mice that received the sham operation. **e**, **f** The influence of miR-325-3p dysregulation on the expression of RIPK3 mRNA (**e**) and protein (**f**) in MI mice. **P < 0.01 compared to MI mice treated with agomiR-control. MI, myocardial infarction; agomiR-325-3p, miR-325-3p agomir; antagomiR-325-3p, miR-325-3p antagomir; agomiR-control, scrambled agomir or antagomir control; RIPK3, receptor-interacting serine/threonine protein kinase 3
